# Disruption of splicing-regulatory elements using CRISPR/Cas9 to rescue spinal muscular atrophy in human iPSCs and mice

**DOI:** 10.1093/nsr/nwz131

**Published:** 2019-09-03

**Authors:** Jin-Jing Li, Xiang Lin, Cheng Tang, Ying-Qian Lu, Xinde Hu, Erwei Zuo, He Li, Wenqin Ying, Yidi Sun, Lu-Lu Lai, Hai-Zhu Chen, Xin-Xin Guo, Qi-Jie Zhang, Shuang Wu, Changyang Zhou, Xiaowen Shen, Qifang Wang, Min-Ting Lin, Li-Xiang Ma, Ning Wang, Adrian R Krainer, Linyu Shi, Hui Yang, Wan-Jin Chen

**Affiliations:** 1 Department of Neurology and Institute of Neurology, The First Affiliated Hospital of Fujian Medical University, Fuzhou 350005, China; 2 Fujian Key Laboratory of Molecular Neurology, Fujian Medical University, Fuzhou 350005, China; 3 Institute of Neuroscience, State Key Laboratory of Neuroscience, Key Laboratory of Primate Neurobiology, CAS Center for Excellence in Brain Science and Intelligence Technology, Shanghai Institutes for Biological Sciences, Chinese Academy of Sciences, Shanghai 200031, China; 4 Key Lab of Computational Biology, CAS-MPG Partner Institute for Computational Biology, Shanghai Institutes for Biological Sciences, Chinese Academy of Sciences, Shanghai 200031, China; 5 Department of Anatomy, Histology & Embryology, Shanghai Medical College, Fudan University, Shanghai 200032, China; 6 Cold Spring Harbor Laboratory, Cold Spring Harbor, New York 11724, USA

**Keywords:** spinal muscular atrophy, *SMN2*, splicing-regulatory elements, CRISPR/Cas9, germline correction

## Abstract

We here report a genome-editing strategy to correct spinal muscular atrophy (SMA). Rather than directly targeting the pathogenic exonic mutations, our strategy employed Cas9 and guide-sgRNA for the targeted disruption of intronic splicing-regulatory elements. We disrupted intronic splicing silencers (ISSs, including ISS-N1 and ISS + 100) of survival motor neuron (SMN) 2, a key modifier gene of SMA, to enhance exon 7 inclusion and full-length SMN expression in SMA iPSCs. Survival of splicing-corrected iPSC-derived motor neurons was rescued with SMN restoration. Furthermore, co-injection of Cas9 mRNA from *Streptococcus pyogenes* (SpCas9) or Cas9 from *Staphylococcus aureus* (SaCas9) alongside their corresponding sgRNAs targeting ISS-N1 into zygotes rescued 56% and 100% of severe SMA transgenic mice (*Smn*^−/−^, *SMN2*^tg/−^). The median survival of the resulting mice was extended to >400 days. Collectively, our study provides proof-of-principle for a new strategy to therapeutically intervene in SMA and other RNA-splicing-related diseases.

## INTRODUCTION

The majority of human protein-coding genes are able to undergo alternative pre-mRNA splicing and pathogenic mutations that affect splicing are prevalent [1]. The pre-mRNA splicing process involves multiple interactions between pre-mRNA molecules including *cis*-acting sequences present in the pre-mRNAs that are known as splicing-regulatory elements (SREs), small nuclear ribonucleoproteins and splicing-factor proteins [2]. Dysregulated splicing drives the pathogenesis of multiple diseases, including familial dysautonomia, cystic fibrosis, tau-related disease and spinal muscular atrophy (SMA) [3–6].

SMA is the most common inherited cause of infant mortality globally [7]. More than 98% of SMA patients are homozygous for the deletion of the survival motor neuron 1 (*SMN1*) gene [8]. Interestingly, it is known that the survival motor neuron 2 (*SMN2*) gene (an almost identical copy of *SMN1*) is not able to fully compensate for the lack of *SMN1* (and its functional gene product: full-length survival motor neuron (SMN) protein (SMN-FL)) in SMA patients; this is because, compared to *SMN1*, *SMN2* has a translationally silent C-to-T transition at position 6 in its seventh exon that, via alternative splicing, causes more than 90% of the SMN protein produced by cells to be truncated and thus non-functional [9,10]. Specifically, this C-to-T transition changes an exonic splicing enhancer into an exonic splicing silencer by destroying a binding site for the pre-mRNA-splicing factor SF2/ASF (aka SRSF1) and simultaneously creating a binding site for the nuclear ribonucleoprotein hnRNPA1 that functions in pre-mRNA processing [11,12]. It is well established that both hnRNPA1 and hnRNPA2 can bind to two SREs located in intron 7 of *SMN2*: ISS (intronic splicing silencer)-N1 and ISS + 100 [13–15]. This binding, which helps in the fine-tuning of the repression of exon 7 splicing, has pathomechanistic consequences in SMA [16].

The CRISPR/Cas9 RNA-guided genome engineering system, a Type II CRISPR-Cas bacterial adaptive immune system, has great potential for correcting disease-causing mutations [17–19]. Cas9 from *Streptococcus pyogenes* (SpCas9) or *Staphylococcus aureus* (SaCas9) can be directed by single-guide RNAs (sgRNAs) to specific genomic loci, where they induce double-strand breaks (DSBs) adjacent to so-called protospacer-adjacent motifs (PAMs) (NGG for SpCas9 and NNGRRT for SaCas9). DSBs are then resolved by either the non-homologous end-joining (NHEJ) repair pathway or the homology-directed repair (HDR) pathway: NHEJ introduces additional insertions or deletions (indels) at the DSBs; HDR can ‘correct’ a mutant allele by replacing the original sequence with a supplied exogenous DNA molecule [20]. It is widely accepted that NHEJ-based genome editing is more convenient and efficient than HDR-based methods [21,22].

Here, we report our successful development of a genome-editing strategy for the Cas9-mediated disruption of SREs as an alternative to adopting the typical genome-editing approach of targeting pathogenic exonic mutations. Our results showed that CRISPR/Cas9-based disruption of two *SMN2* SREs (ISS-N1 and ISS + 100) rescued the SMA phenotypes in human induced pluripotent stem cells (iPSCs) and in germline-corrected SMA mice. We remarkably enhanced *SMN2* exon 7 inclusion rates and increased SMN-FL expression in SMA patient-derived iPSCs and motor neurons (MNs). Furthermore, 62.8% of the severe SMA mice (*Smn*^−/−^, *SMN2*^tg/−^) could be rescued by zygote co-injection of SpCas9 or SaCas9 mRNA and their corresponding sgRNAs targeting ISS-N1. The median lifespan of the germline-corrected SMA mice markedly increased to >400 days, with a maximum lifespan of >600 days.

## RESULTS

### Alternative splicing is corrected in SMA iPSCs with *SMN2*-ISSs disruption

**Figure 1.**
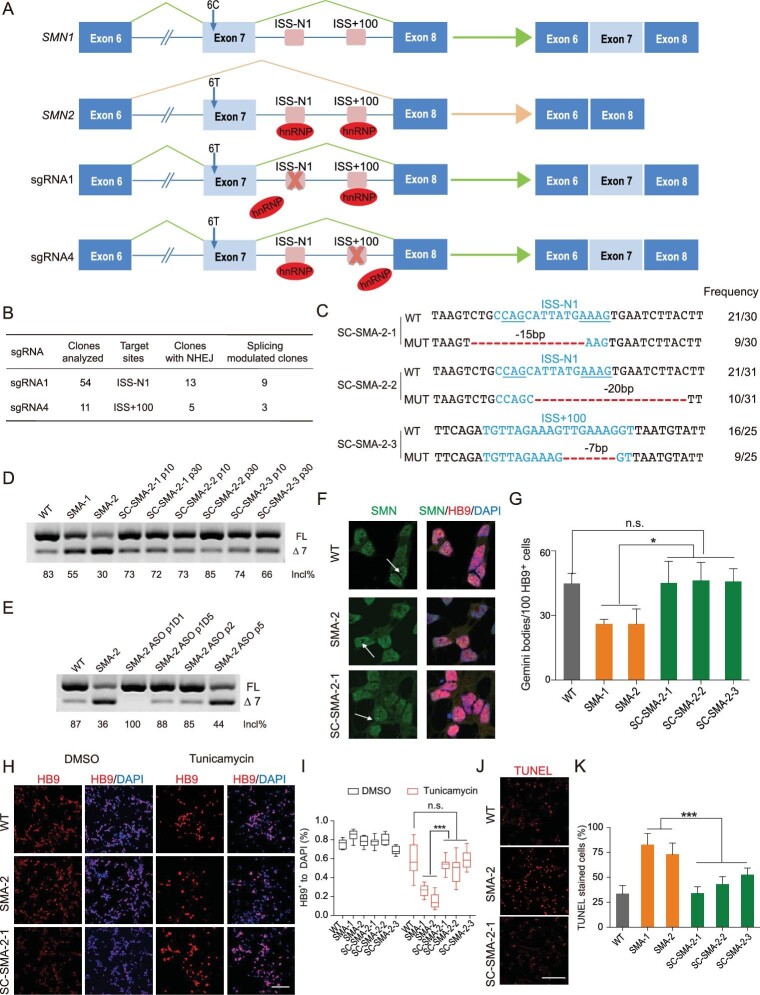
Alternative splicing is modulated in ISS-N1 and ISS + 100 disrupted SMA iPSCs and motor neurons. (A) Schematic of CRISPR/Cas9-mediated disruption of ISS-N1 and ISS + 100 in intron 7 of *SMN2*. (B) NHEJ and splicing-modulation efficiencies by targeting ISS-N1 and ISS + 100. (C) Alignments of corrected sequences from SMA iPSCs with Cas9-sgRNA-mediated disruption at the ISS-N1 and ISS + 100 loci. The deletions are indicated by a red dashed line. Blue lines indicate the two core motif sequences (CAG and AAAG) of ISS-N1. The column on the right indicates the percent of the relevant genotype in total sequencing reads. (D) RT-PCR analysis of SMN2 mRNA in SMA patient-derived iPSCs. The SC-SMA iPSCs were evaluated at passages (p) 10 and 30. Incl% = (FL /(FL + Δ7)) × 100. (E) RT-PCR analysis of SMN2 mRNA in iPSCs. The ASO-treated SMA-2 iPSCs were assessed at p1(Day (D) 1), p1(D5), p2 and p5. (F) Nuclear Gemini body localization in motor neurons co-stained with SMN and HB9 antibodies. Gemini bodies are indicated by arrows. Scale bar, 10 μm. (G) Quantification of SMN^+^ Gemini bodies in HB9^+^ MNs at Day 13; *n* = 3, with about 100 cells examined per group. (H) MNs were treated with DMSO or 40 μM tunicamycin for 38 h. HB9 was used to mark surviving MNs, counterstained with DAPI. Scale bar, 100 μm. (I) Quantification (ratio of surviving MNs to total cells) of cell viability after tunicamycin treatment; cell viability was increased in SC-SMA-iPSCs-derived MNs; *n* = 3. (J) Treated MNs were assayed with TUNEL to mark apoptotic cells. Scale bar, 100 μm. (K) Quantification of dead MNs to total cells after tunicamycin treatment. The extent of MN apoptosis was reduced in SC-SMA-iPSCs-derived MNs; *n* = 3. Error bars indicate means ± SDs. ns, not significant; ^*^P < 0.05; ^*^^*^^*^P < 0.001; one-way ANOVA.

Our strategy for disrupting the *SMN2*-ISSs in human SMA iPSCs was based on three sgRNAs (sgRNA1, 2 and 3) that we designed to target ISS-N1 and one sgRNA (sgRNA4) to target ISS + 100 (Fig. 1A and Supplementary Fig. 1A). We generated iPSCs from fibroblasts of a wild-type (WT) control and an SMA patient with 2 *SMN2* copies (termed SMA-1) using a non-integrating Sendai virus. We obtained an additional SMA iPSC line with three *SMN2* copies (termed SMA-2) from Coriell Cell Repositories (Supplementary Fig. 1B). All iPSCs displayed human embryonic stem-cell (hESC) morphology, expressed characteristic pluripotency markers (AP, NANOG, OCT4, SOX2, TRA-1–60 and SSEA-4) (Supplementary Fig. 2A), exhibited normal karyotypes (Supplementary Fig. 2B) and generated teratomas *in vivo* (Supplementary Fig. 2C).

Plasmids expressing Cas9 and each of the four sgRNAs were separately transfected into SMA-2 to test the cutting efficiency of each sgRNA. A T7 endonuclease I (T7EI) assay, which specifically cleaves heteroduplexes formed by the hybridization of WT and mutant DNA sequences, revealed that sgRNA1 and sgRNA4 target ISSs, with 5.28% and 2.14% efficiency, respectively. In contrast, no DSBs were detected with sgRNA2 or sgRNA3 (Supplementary Fig. 3A), so we used sgRNA1 targeting ISS-N1 and SpCas9 vectors for transfection of SMA-2 iPSCs followed by an enhanced green fluorescence selection protocol. Screening of 54 clones revealed that 13 (24.1%) of the splicing-corrected SMA-2 clones showed NHEJ, of which 9 (69.2%) showed the desired splicing correction (Fig. 1B). To test the efficiency of sgRNA4 in disrupting ISS + 100, we further transfected SMA-2 iPSCs with a plasmid that contained both Cas9 and sgRNA4, and found that 45% (5/11) of clones showed NHEJ, of which 60% (3/5) showed the desired splicing correction (Fig. 1B).

Subsequently, we used sequencing and reverse-transcription polymerase chain reaction (RT-PCR) to examine associations between particular clonal genotypes and the extent of full-length SMN2 (SMN2-FL) mRNA expression. The SMN2-FL mRNA expression level was strongly promoted among the splicing-corrected iPSCs (termed SC-SMA) (Fig. 1C and D). Moreover, RT-PCR analysis revealed that only one of the three SMN loci harbored an edited ISS-N1 or ISS + 100, which was sufficient to correct splicing (Fig. 1C and D). Immunoblotting results also confirmed that SMN protein expression was significantly increased in splicing-corrected clones (Supplementary Fig. 3B and C). We also confirmed the splicing correction of Cas9-mediated SREs disruption in SMA-1 iPSCs (Supplementary Fig. 3D–F).

We next treated unedited SMA-2 iPSCs with an antisense morpholino oligonucleotide (MO-10–29) [23] targeting ISS-N1, which increased the exon 7 inclusion rate to 100%, but this rate quickly decreased to the level of untreated cells with continual passaging (Fig. 1E). This compares to the permanent effects of disrupting an SRE by our strategy. Moreover, our genome-edited iPSCs retained uniform expression of pluripotency markers (Supplementary Fig. 4A), karyotype stability (Supplementary Fig. 4B) and the ability to generate teratomas *in vivo* (Supplementary Fig. 4C). We used Digenome-seq (*in vitro* Cas9-digested whole-genome sequencing) to further measure the genome-wide Cas9 off-target activity of our sgRNAs in SMA iPSCs [24,25]. A total of 29 potential off-target sites (1 for sgRNA1 and 28 for sgRNA4) throughout the genomes of multiple iPSCs clones were captured in an unbiased manner using Digenome-seq and none of the potential regions sequenced showed off-target cleavage (Supplementary Table 1). To further confirm the specificity of these sgRNAs on a genome-wide scale, we also performed high-throughput mRNA sequencing on the splicing-corrected iPSCs (SC-SMA-2-2 and SC-SMA-2-3) generated using sgRNA1 or sgRNA4. Neither of the two iPSCs showed detectable off-target effects (Supplementary Fig. 5), highlighting that sgRNA1- and sgRNA4-guided gene targeting and splicing regulation are apparently highly specific editing processes.

### SMN restoration relieves the degeneration of SMA iPSCs-derived MNs

We then used a modified version of a previously described cell-culture protocol to differentiate the splicing-corrected iPSCs lines into MNs [26,27]. We successfully differentiated one WT iPSCs, two SMA iPSCs (SMA-1 and SMA-2) and three established isogenic SC-SMA-2 iPSCs into spinal MNs (Supplementary Fig. 6). Unlike the unedited control MNs, the MNs derived from splicing-corrected SMA iPSCs had many Gemini bodies—nuclear aggregate structures formed by SMN (Fig. 1F and G). These findings suggest that the disruption of *SMN2*-ISSs can restore SMN expression in SC-SMA-iPSCs-derived MNs.

The SMN protein has minimal anti-apoptotic effects and SMN depletion activates a high basal level of endoplasmic reticulum (ER) stress signaling to cause apoptosis in MNs [28]. To investigate whether the increased SMN protein levels resulting from our editing strategy reduce MN sensitivity to exogenous ER stress, we treated all of our iPSCs-derived MNs for 38 h with 40 μM tunicamycin—a compound that induces ER stress [29]. We observed that SMA MNs were more vulnerable to tunicamycin treatment (∼75% MNs loss, *P* < 0.001) than WT cultures (∼35% MNs loss) and than SC-SMA-2 cultures (∼42% MNs loss) (Fig. 1H and I). However, there were no significant differences among dimethyl sulfoxide (DMSO)-treated control cultures (*P* > 0.1, Fig. 1H and I). Next, we assessed the extent of apoptosis using a TUNEL assay after tunicamycin treatment. Increased apoptosis was seen in SMA MN cultures as compared with WT and SC-SMA-2 cultures (*P* < 0.001), with about 78% of SMA, 33% of WT and 43% of SC-SMA-2 cultures stained positively for TUNEL (Fig. 1J and K). Overall, these results establish that increasing SMN levels can ameliorate the degeneration of SMA MNs.

### CRISPR/Cas9-mediated disruption of *SMN2* ISS-N1 in mice germline DNA prevents SMA

**Figure 2.**
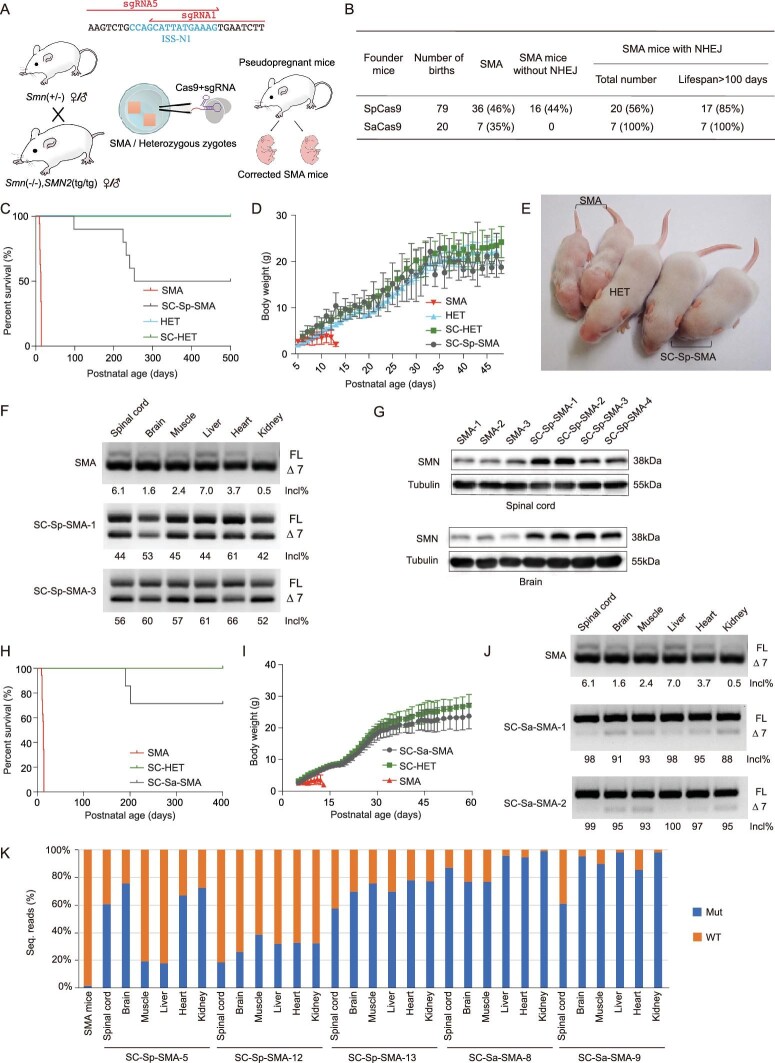
Disruption of ISS-N1 alleviated SMA-associated phenotypes in splicing-corrected SMA mice. (A) Strategy of the CRISPR/Cas9-mediated disruption of ISS-N1 in SMA mice via germline gene therapy. (B) NHEJ and survival efficiencies in SC-SMA mice. (C) Kaplan–Meier survival curves for SC-Sp-SMA mice edited by SpCas9-sgRNA (*n* = 10), SMA mice (*n* = 35), heterozygous (HET) mice (*n* = 10) and SC-HET mice (*n* = 10). (D) Body weight was assessed from P6 to P48 for SMA (*n* = 15), SC-Sp-SMA (*n* = 20), HET (*n* = 8) and SC-HET (*n* = 19) mice. (E) Two SC-Sp-SMA mice were similarly sized with their heterozygous littermates. (F) RT-PCR analysis of SMN2 mRNA in multiple tissues from SMA and SC-Sp-SMA mice. (G) Immunoblot analysis of SMN protein in spinal-cord and brain samples from SMA and SC-Sp-SMA mice. (H) Kaplan–Meier survival curves for SC-Sa-SMA mice disrupted by SaCas9-sgRNA (*n* = 7) and SC-HET mice (*n* = 12). Data from SMA mice were the same as in (C). (I) Body weight was assessed from P5 to P59 for SC-Sa-SMA (*n* = 7) and SC-HET (*n* = 12) mice. Data from SMA mice were the same as in (D). (J) RT-PCR analysis of SMN2 mRNA in multiple tissues from SMA and SC-Sa-SMA mice. (K) Editing efficiency in different organs of the edited mice (lifespan >400 days). Wild-type (WT) reads without mutations are represented by orange bars; mutant reads of ISS-N1 are represented by blue bars. Mut, mutant. Error bars indicate means ± SDs.

Having thus established that our strategy worked as designed and successfully rescued disease symptoms in both iPSCs and MNs, we next investigated whether an SMA disease model mouse (*Smn*^−/−^; *SMN2*^tg/−^) [30] could be rescued by the disruption of the *SMN2*-ISSs. SpCas9 mRNA and sgRNA1 targeting ISS-N1, the most common target for treating SMA [31], were co-injected into the cytoplasm of zygotes (harvested from heterozygous BH mice (*Smn*^+/−^) that had been previously mated with type III HF mice (*Smn*^−/−^; *SMN2*^tg/tg^)) (Fig. 2A). Furthermore, sgRNA1 targeting caused no obvious deleterious effect on the birth rate of ISS-N1-disrupted mice compared with control non-ISS-N1 sgRNA targeted mice (injected with non-ISS-N1 sgRNA in our laboratory) (Supplementary Fig. 7A). Of the 36 live SMA pups born, 20 had NHEJ edits at the *SMN2* ISS-N1 locus and 85% (17/20) of these were rescued for SMA disease symptoms and had lifespans that reached >100 days (Fig. 2B). Moreover, the splicing-corrected SMA (SC-Sp-SMA) mice showed a significant improvement in median lifespan to >400 days (SC-Sp-SMA-5∼14^#^); this was only 13 days for unedited control SMA mice (Fig. 2C and Supplementary Table 2). So far, 58.3% of the SC-Sp-SMA mice remain alive and the maximum survival time has reached 600 days. Of note, the body weight of SC-Sp-SMA mice was elevated (Fig. 2D). At postnatal Day (P) 9, most SC-Sp-SMA mice were comparable in size to their littermate control mice (Fig. 2E).

We performed righting-reflex and grip-strength tests to further assess the motor function of SC-Sp-SMA mice. Compared with non-corrected SMA mice, SC-Sp-SMA mice were faster in righting up at P11 (Supplementary Fig. 7B). To evaluate the muscle strength of SC-Sp-SMA mice, forelimb grip-strength analysis was carried out from P23 to P33. Because of their short lifespan (∼13 days), the non-corrected SMA mice were unsuitable for inclusion in this experiment. We therefore compared our SC-Sp-SMA mice with heterozygous littermate mice (*Smn*^+/−^; *SMN2*^tg/−^, termed SC-HET mice) and observed no significant differences in strength at P33 (Supplementary Fig. 7C).

We next examined SMN expression in six different organs of SC-Sp-SMA mice at P9. RT-PCR analysis showed that, compared to SMA mice, all of the tested organs of the SC-Sp-SMA mice had increases in their exon 7 inclusion rates (∼50% vs ∼7%) (Fig. 2F) and immunoblotting confirmed the increased accumulation of the SMN protein (Fig. 2G). Note that we did observe considerable variation in the SMN levels among the four SC-Sp-SMA mice that we examined; genotyping analysis revealed that this variation could be explained by differences in the sequences produced by NHEJ editing in the different progeny (Supplementary Fig. 7D and Supplementary Table 2), with the highest levels of SMN resulting from edits to initial CAG or AAAG motifs.

In addition to the SC-Sp-SMA mice, we also generated splicing-corrected mice by co-injecting SaCas9 mRNA and sgRNA5 into zygotes. This produced seven live SC-Sa-SMA pups that all had disruption of *SMN2* ISS-N1 and that had significant increases in their median survival times (>400 days, SC-Sa-SMA-3∼9^#^) and body weights (Fig. 2H and I, and Supplementary Table 3). We did observe variation in the lifespan of SC-Sa-SMA mice; genotyping analysis revealed that this variation could be explained by differences in the sequences produced by NHEJ editing (Supplementary Fig. 7D and Supplementary Table 3) and mice with the CAG or AAAG motif edited exhibited the longest lifespan. RT-PCR analysis indicated that alternative splicing of SMN2 mRNA was corrected in these pups. Compared to SMA mice at P9, the SC-Sa-SMA mice exhibited increases in the exon 7 inclusion rate in multiple tissues (∼95% vs ∼7%) (Fig. 2J).

It is widely appreciated that germline-edited mice frequently exhibit mosaicism [32,33]. We therefore conducted an experiment in which we sampled six different organs from three SC-Sp-SMA mice and two SC-Sa-SMA mice (all their lifespans >400 days) to quantify WT and mutant allele indel frequencies in different lines: deep-sequencing analysis showed variable distributions of post-edit genotypes. The correction rates for *SMN2* in different organs from genetically mosaic SC-SMA mice ranged widely, from 18% to 90% (Fig. 2K). We used Digenome-seq to examine off-target effects in the SMA mice genome and observed no potential off-target mutations for sgRNA1 and only one for sgRNA5; we further examined this putative off-target mutation in the spinal cord and muscle of SC-SMA mice using deep sequencing and no such mutations were found in genomic DNA from these tissues (Supplementary Table 4). These results indicate that our strategy of disrupting ISS-N1 is an effective method for rescuing SMA phenotypes in mice.

**Figure 3.**
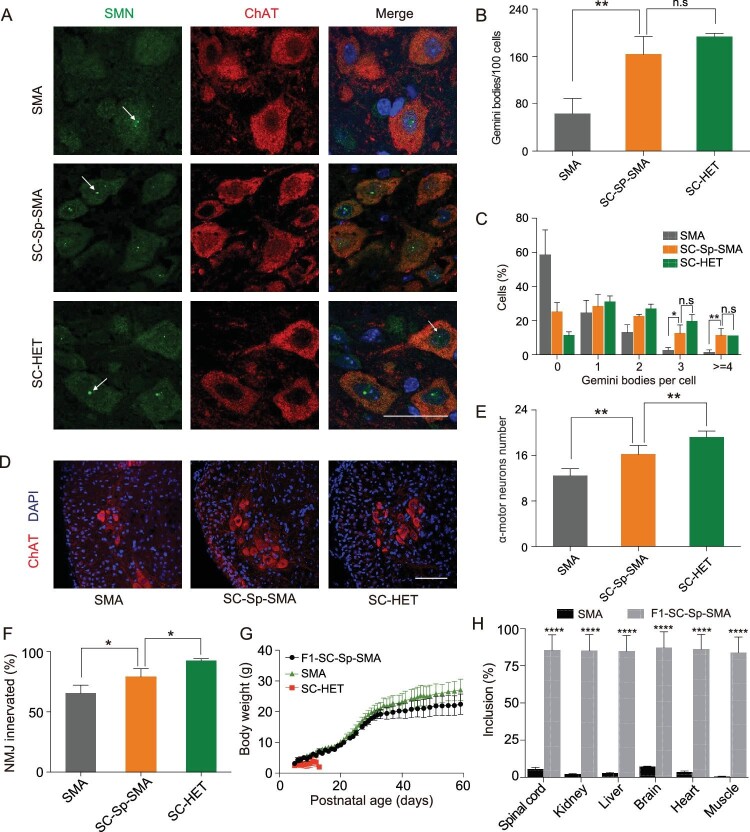
SMN restoration alleviates spinal MN degeneration and NMJ denervation in SC-Sp-SMA mice. (A) Nuclear Gemini bodies localization in spinal-cord L1–L2 motor neurons was determined by co-staining with SMN and ChAT antibodies. Nuclei were counterstained with DAPI. Gemini bodies are indicated by arrows. Scale bar, 50 μm. (B) and (C) Quantification of Gemini bodies per 100 motor neurons (B) and percentage of motor neurons containing zero, one, two, three, four or more Gemini bodies (C) in SMA (*n* = 3), SC-Sp-SMA (*n* = 6) and SC-HET (*n* = 3) mice. (D) Representative images of spinal-cord L1–L2 ventral-horn ChAT^+^ MNs (red) in the above three groups. Scale bar, 100 μm. (E) MNs labeled in (D) were counted. SMA (*n* = 6), SC-Sp-SMA (*n* = 5) and SC-HET (*n* = 6). (F) Quantification of the innervated NMJs in SMA (*n* = 3), SC-Sp-SMA (*n* = 5) and SC-HET (*n* = 4) mice. (G) Body weight was assessed from P5 to P59 for F1-SC-Sp-SMA (*n* = 9). Data from SMA mice and SC-HET were the same as in Figure 2 (I). (H) Statistical analysis of SMN2 mRNA in multiple tissues from SMA and F1-SC-Sp-SMA mice. Error bars indicate means ± SDs. ns, not significant; ^*^P < 0.05; ^*^^*^P < 0.01; ^*^^*^^*^^*^P < 0.0001; one-way ANOVA.

We subsequently performed immunohistochemical analysis of Gemini body number, MN counts and neuromuscular-junction (NMJ) innervation patterns in SC-Sp-SMA mice. A striking elevation in the proportion of cells containing Gemini bodies was observed in SC-Sp-SMA mice compared with untreated SMA mice (Fig. 3A–C). Moreover, the extent of the spinal MN degeneration and NMJ denervation was significantly reduced in SC-Sp-SMA mice as compared to untreated SMA mice (Fig. 3D–F and Supplementary Fig. 7E).

SMN function was restored and inherited in the progeny of rescued SMA mice.

We next assessed reproductive ability after disruption of ISS-N1. The founder SC-Sp-SMA female mice were crossbred to heterozygous BH (*Smn*^+/−^) male mice. These crosses generated nine homozygous F1 SC-Sp-SMA mice and one unedited SMA mouse (Supplementary Fig. 7F and G). All of the F1 SC-Sp-SMA pups had normal lifespans and body weights (Fig. 3G and Supplementary Fig. 7G). RT-PCR and immunohistochemistry analyses showed that the F1 SC-Sp-SMA pups had stronger SMN expression than the SMA mice (Fig. 3H and Supplementary Fig. 7H) and had significantly increased numbers of MNs and properly innervated NMJs (Supplementary Fig. 7I and J). Thus, SMN function was effectively restored and inherited in the progeny of SC-Sp-SMA mice.

## DISCUSSION

We induced Cas9-mediated SREs disruption in SMA patient-derived iPSCs and in mouse zygotes with high efficiency. *In vitro*, SMN2 splicing correction and SMN-FL restoration were seen once one copy of the CAG or AAAG motif had been disrupted in *SMN2*-ISSs of SMA iPSCs. Notably, MNs differentiated from SC-Sp-SMA iPSCs exhibited significantly reduced apoptosis. *In vivo*, SMA mice rescued by germline *SMN2*-ISSs disruption exhibited enhanced lifespan, body weight and motor functioning, as well as significantly increased numbers of MNs and properly innervated NMJs.

Several aspects of this new strategy merit consideration. First, by targeting an intronic splicing element rather than exonic sequences, our approach dramatically reduces the likelihood of inducing pathogenic frameshift mutations in the protein-coding sequence of the targeted gene. Second, considering the widely appreciated fact that NHEJ-mediated disruption of SREs is more efficient than HDR-mediated mutation replacements, it is reasonable to anticipate that the disruption edits needed in our approach are easier to generate than precise in-frame exonic edits. It is also notable that no exogenous DNA template is needed with our SREs disruption strategy. With future discoveries and engineering of additional Cas9 species or other endonucleases that generate DSBs, it is likely that potentially every SRE can be precisely targeted for disruption [34–37]. Importantly, although our current analysis focused only on two SREs of the *SMN2* gene, similar approaches are should be applicable for other SREs, thereby substantially broadening the applicability of this technology for therapeutic interventions.

Antisense oligonucleotide(s) (ASO) hold promise for the treatment of multiple neurodegenerative diseases. Nusinersen (Spinraza™), an ASO masking ISS-N1, is the first approved SMA therapeutic for pediatric and adult patients [38]. Despite its long half-life in the central nervous system (CNS), it requires four loading doses, followed by three annual maintenance doses, subjecting patients to repeated intrathecal injections [39]. Importantly, as NHEJ-mediated disruption of SREs induces a permanent splicing correction, our strategy obviates the need for continual administration of ASOs. The recently US Food and Drug Administration-approved adeno-associated virus (AAV) serotype 9 (AAV9) carrying SMN complementary DNA encoding the missing SMN protein, AVXS-101 (Zolgensma^®^), is a single-dose gene-replacement therapy for SMA. As such single-dose correction is not always effective and it is unmanageable to keep injecting the virus, treatment with Spinraza remains as an option to treat those failures [40,41].

SaCas9 has been shown to mediate genome editing *in vivo* with high efficiency and specificity [42]. We also found that SaCas9-mediated *SMN2*-ISSs editing was especially successful, achieving a high-yield generation of embryos carrying the ablated ISS-N1 sequences. We did observe variation in lifespan of germline-corrected SMA mice using SpCas9 and SaCas9, which could be explained by differences in the sequences produced by NHEJ editing. To achieve more efficient and specific disruption, the advent of advanced gene-editing tools (e.g. the most recently developed sgRNA indel prediction model—inDelphi) could assist in selecting superior sgRNAs in the future [43]. Any hypothetical targeting of human embryos for editing would require knowledge about which genotypes from each of the parents would suggest an increased probability of producing offspring with a homozygous *SMN1* mutation; however, medical ethics standards prevent human germline editing [44].

Considering variable efficiencies of germline editing on mouse pups, the limited availability of zygotes in humans imposes a major technical challenge in therapeutic applications, besides ethical problems. Previous studies have also revealed the contribution of both cell-autonomous and cell-non-autonomous SMA pathogenesis [45,46]. Moreover, systemic administration (e.g. subcutaneous or intraperitoneal injection) of ASO appears to be more effective than intrathecal or intracerebral

ventricular injection [31,47], suggesting unclear SMN restored cell type(s) that directly contributes to the observed benefit. Considering postnatal correction by CRISPR/Cas9-mediated gene editing, it is also important to determine the efficacy of various delivery options (e.g. AAV, lipid nanoparticle, exosomes) [48,49].

In summary, we have achieved efficient and safe Cas9-mediated *SMN2*-ISSs disruption in SMA iPSCs and mice; this disruption corrects SMN2 splicing errors and restores functional SMN. Despite inconclusive findings regarding the feasibility of our approach in humans, our study establishes a proof-of-principle demonstration for SMA rescue using a strategy based on CRISPR/Cas9-mediated disruption of SREs and thus opens the door for the further development of efficient interventions to treat aberrant splicing diseases such as familial dysautonomia, cystic fibrosis and tau-related disease.

## METHODS

The detailed descriptions of methods are available as Supplementary Materials at *NSR* online.

## Supplementary Material

nwz131_Supplemental_Files
