# Supplementary material for: Disruption of splicing-regulatory elements using CRISPR/Cas9 to rescue spinal muscular atrophy in human iPSCs and mice
Source: Natl Sci Rev. 2019 Sep 3;7(1):92–101. doi: 10.1093/nsr/nwz131 (PMC8446915; doi:10.1093/nsr/nwz131)
Supplement: nwz131_Supplemental_Files [file nwz131_supplemental_files.zip › nwz131_Supplemental_Figures.docx]

**
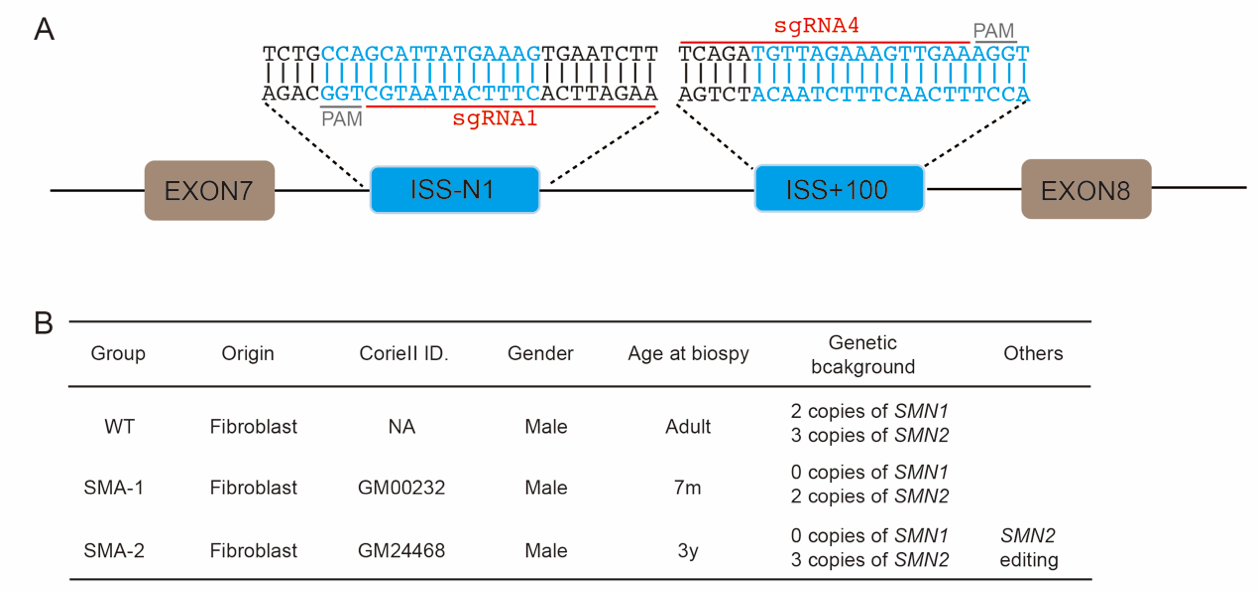
**

**Fig. S1. Schematic diagram of CRISPR/Cas9-mediated SREs disruption strategy and iPSCs information.** (A) Schematic of the targeted ISSs sequences in intron 7 of *SMN2*. The sequences of ISS-N1 and ISS+100 are indicated in blue font. Red lines indicate the sgRNA-targeting sequences. (B) Information of iPS cell lines used in this study was shown. We generated iPSCs from fibroblasts of a wild-type (WT) control and a SMA patient (Coriell ID: GM00232, termed SMA-1) using non-integrating Sendai virus. We also included another SMA iPSC (Coriell ID: GM24468, termed SMA-2).


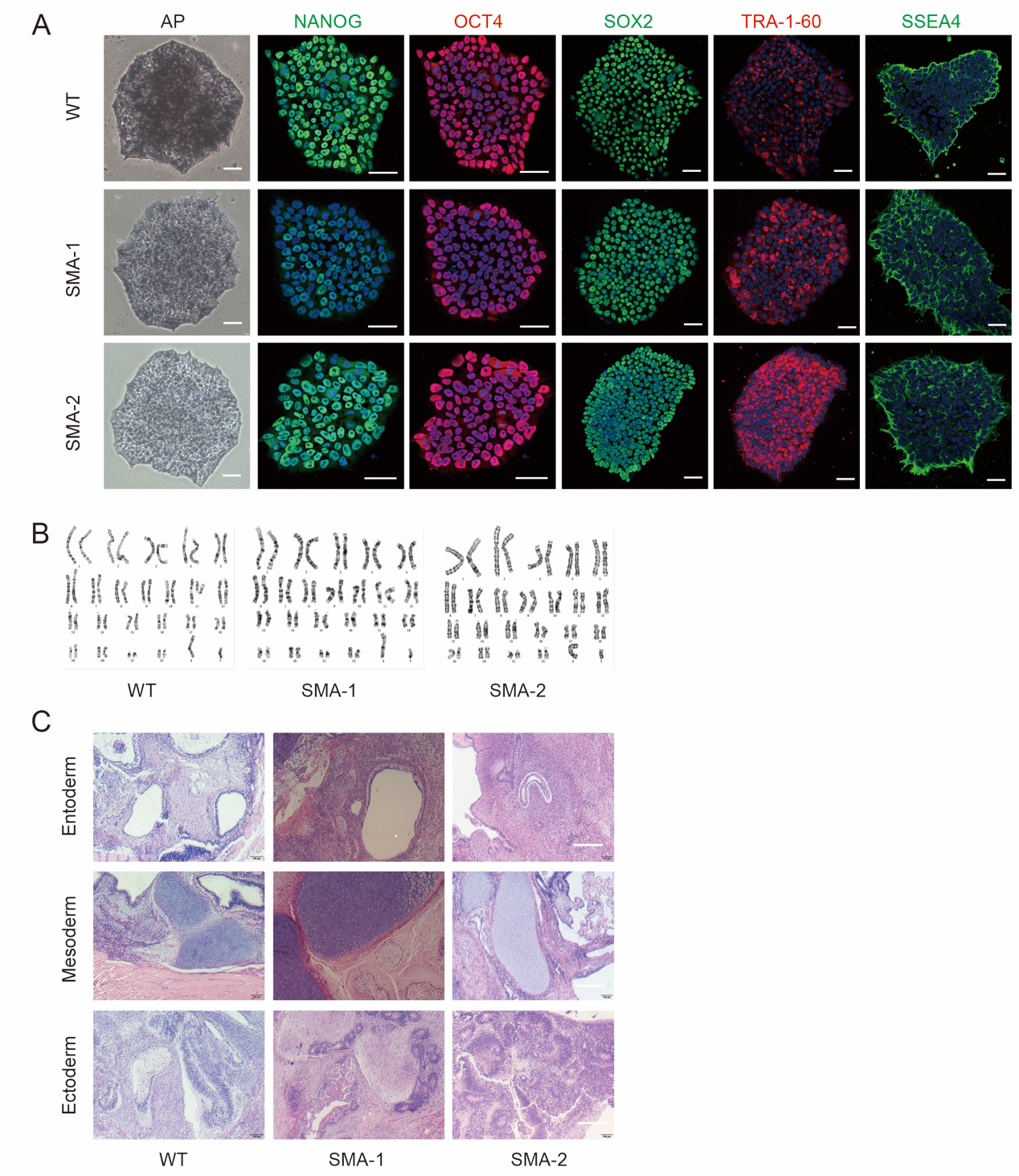


**Fig. S2. Immunostaining, karyotyping and** **teratomas assay of untreated iPSCs.**

(A) Immunostaing of iPSCs showing expression of pluripotency markers: AP, NANOG, OCT4, SOX2, TRA-1-60 and SSEA-4. Scale bar, 50 μm. (B) Karyotyping of iPSCs showing normal karyotypes. (C) HE staining of teratoma showing gut epithelia (endoderm), cartilage (mesoderm) and neural tubes (ectoderm). Scale bar, 250 μm.


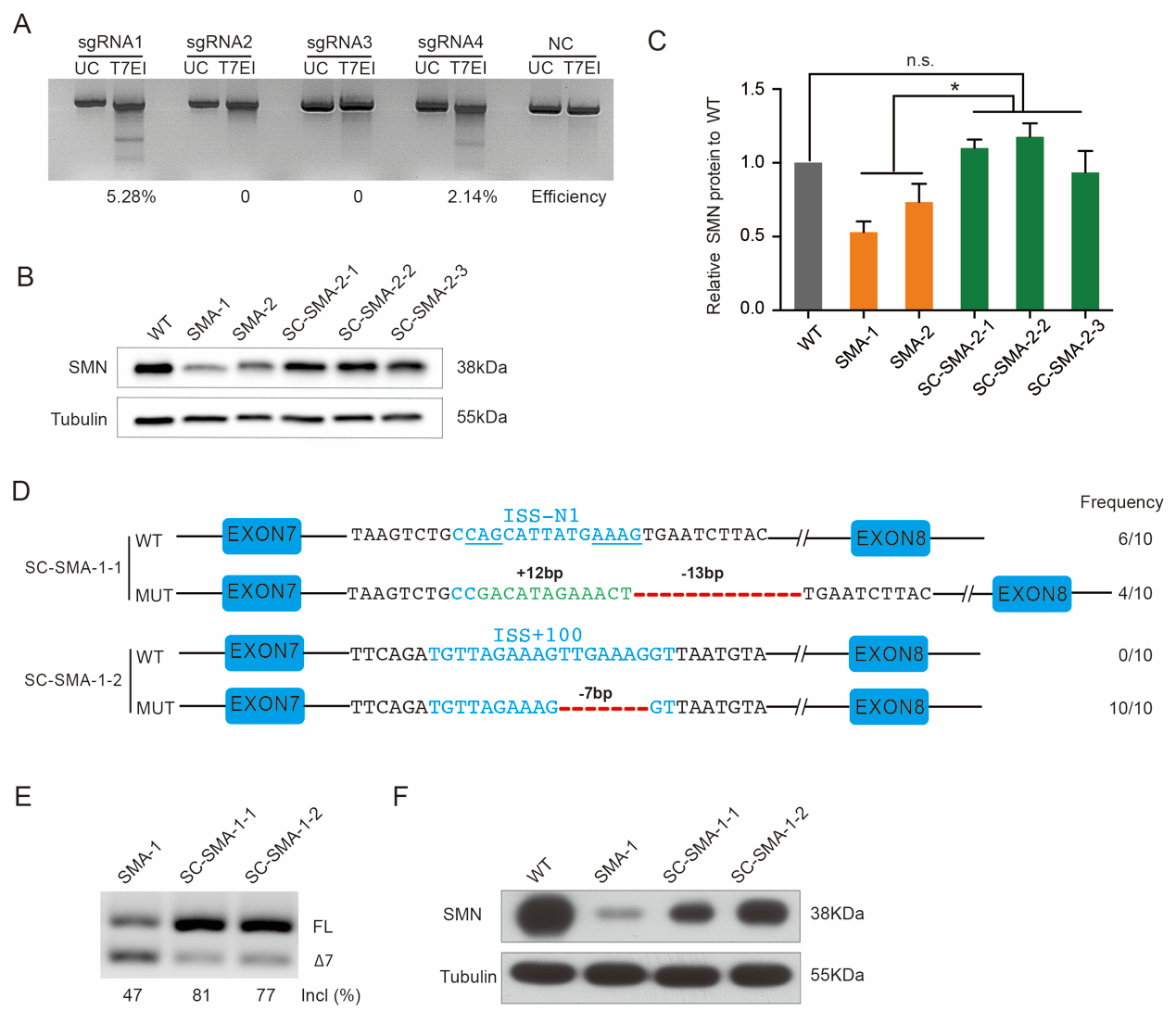


**Fig. S3. Genetic correction of SMA patient-derived iPSCs.**

(A) T7EI assay result of sgRNA 1, 2, 3 and 4. (B) Western blot analysis of SMN protein in SC-SMA-2 iPSCs. (C) Statistical analysis of D (n=3). (*) *P*<0.05 for SC-SMA-2 iPSCs versus unedited SMA iPSCs (SMA-1 and SMA-2), n.s., not significant. Data are presented as mean ± SD. one-way ANOVA. (D) Alignments of corrected sequences from SMA-1 iPSCs with Cas9-sgRNA mediated editing at the ISS-N1 and ISS+100 loci. The deletions are indicated by a red dashed line. Blue lines indicate the two core motif sequences (CAG and AAAG) of ISS-N1. The column on the right indicates the percent of the relevant genotype in total sequencing reads. (E) RT-PCR analysis of SMN2 mRNA in SC-SMA-1 iPSCs. Incl%= (FL/(FL+∆7))$\times$100. (F) Western blot analysis of SMN protein in SC-SMA-1 iPSCs.


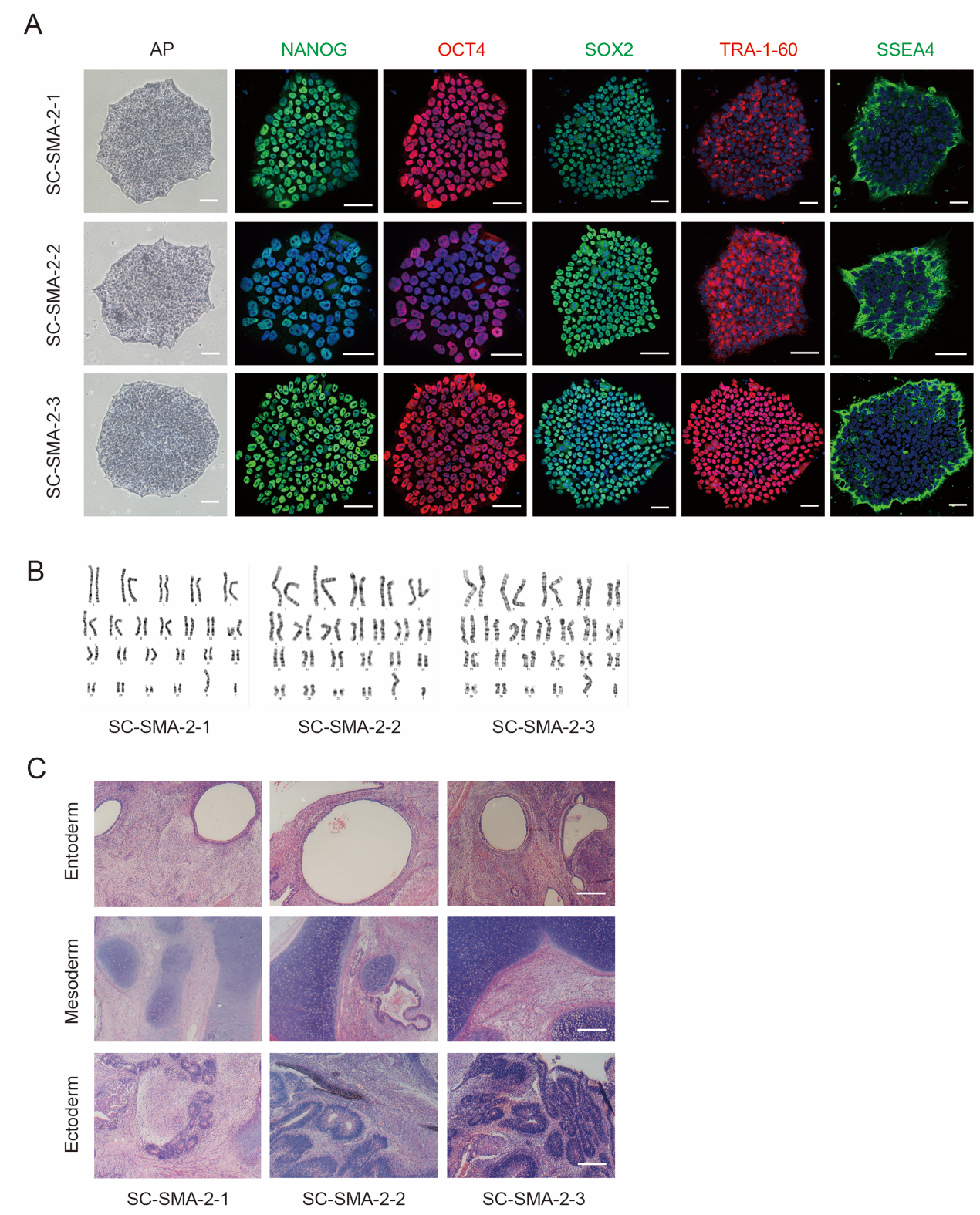


**Fig. S4. Immunostaining, karyotyping and teratomas assay of edited iPSCs.**

(A) Immunostaing of iPSCs showing expression of pluripotency markers: AP, NANOG, OCT4, SOX2, TRA-1-60 and SSEA-4. Scale bar, 50 μm. (B) Karyotyping of iPSCs showing normal karyotypes. (C) HE staining of teratoma showing gut epithelia (endoderm), cartilage (mesoderm) and neural tubes (ectoderm). Scale bar, 250 μm.


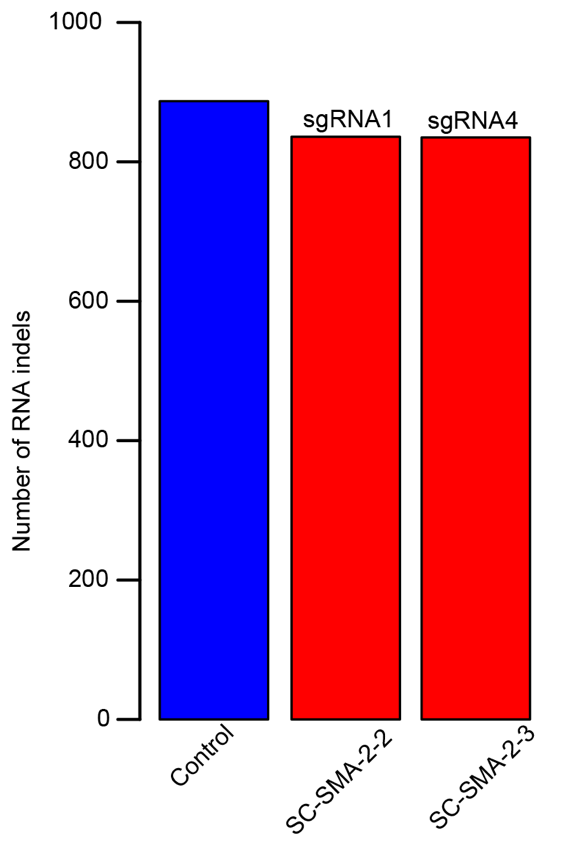


**Fig. S5. The RNA-Seq data of SC-SMA-2-2 (sgRNA1 targeting ISS-N1) and SC-SMA-2-3 (sgRNA4 targeting ISS+100).**

**
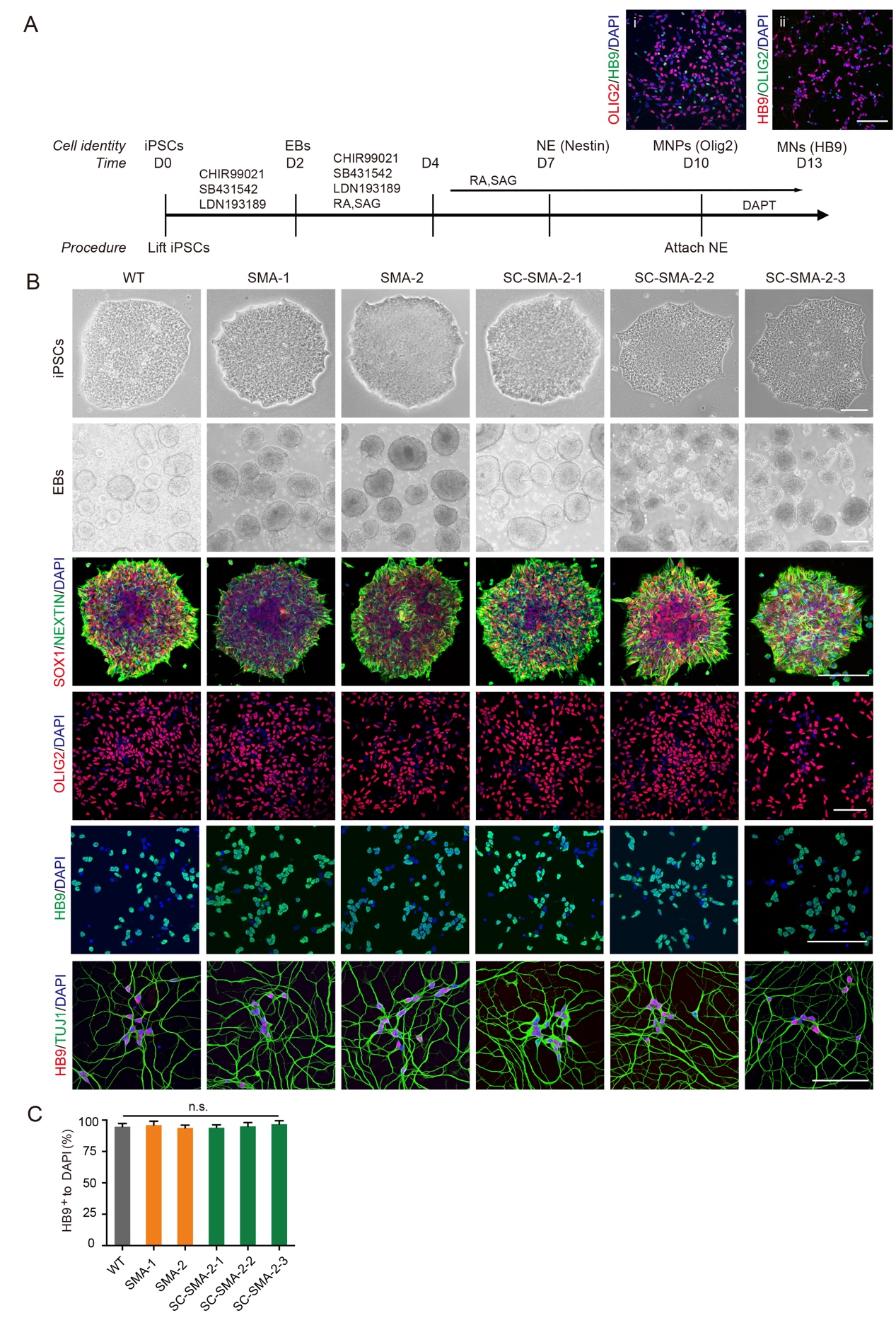
 Fig. S6. Characterization and immunostaining of spinal MNs derived from iPSCs.**

(A) Differentiation scheme of iPSCs to spinal motor neurons. DAPT treatment induces a progressive transition from MNPs (i) to postmitotic MNs (ii). EBs, embryoid bodies; NE, neuroepithelium; MNPs, motor neuron progenitors; MNs, motor neurons. (B) The morphology of iPSCs (at day 0) and EBs (at day 2). Immunostaining of NE (SOX1^+^ and NESTIN^+^ at day 7), MNPs (OLIG2^+^ at day 10), MNs (HB9^+^ and TUJ1^+^ at day 13). Scale bar, 100 μm. (C) Quantification of HB9^+^ MNs in total cells labeled by DAPI at day 13 (n=3). Error bars indicate means ± SD. ns, not significant; one-way ANOVA.


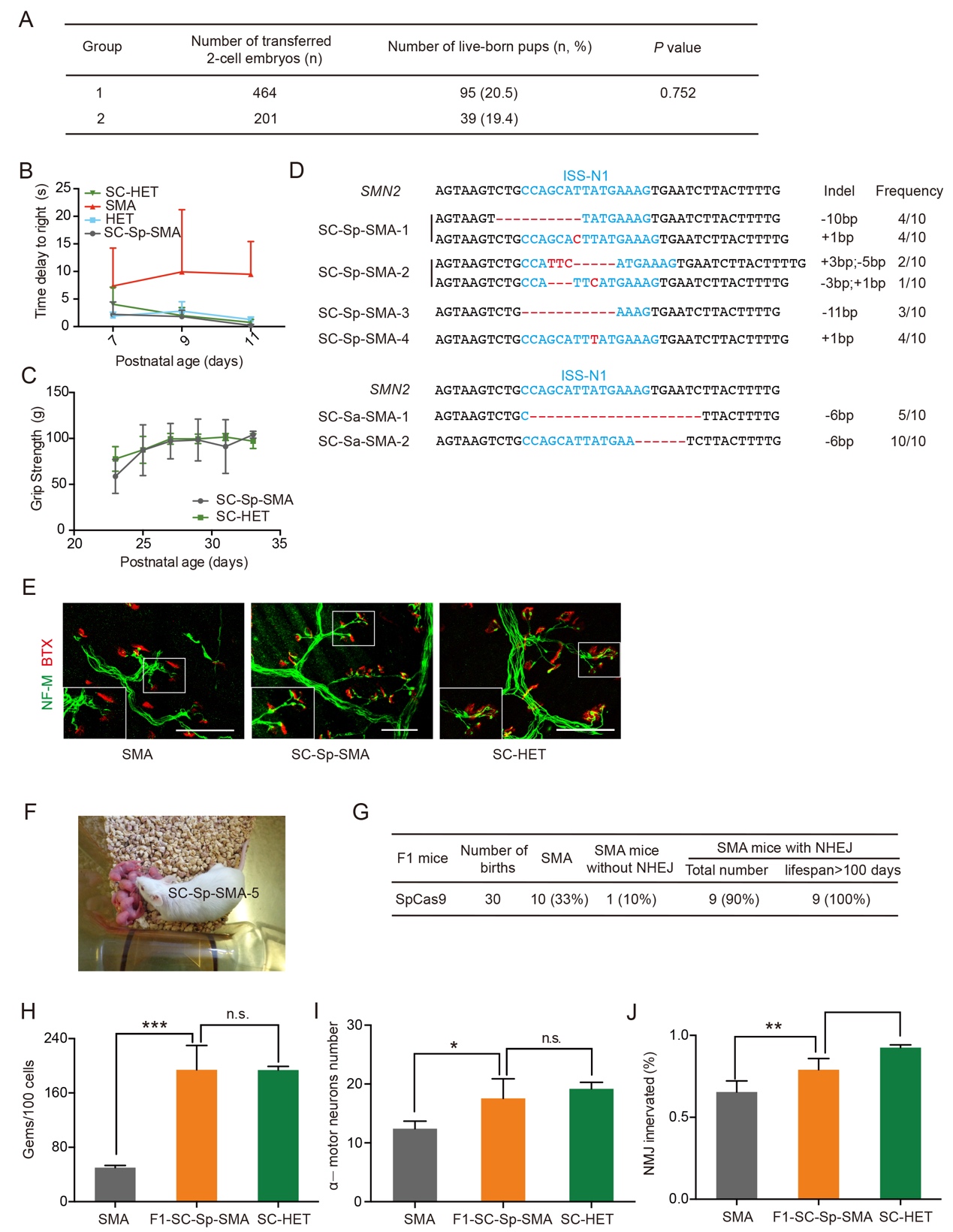


**Fig. S7. SMN function was restored and inherited in the progeny of SC-Sp-SMA mice.**

(A) Birth rate analysis of ISS-N1-sgRNA injected mice. Group 1 is from the current study injected ISS-N1 sgRNA, and Group 2 was from the irrelevant experiments injected non-ISS-N1 sgRNA performed in our laboratory. *P* values were determined by *χ^2^* test. (B) Righting-reflex time was recorded at P7, P9 and P11 for SMA (n=15), SC-Sp-SMA (n=11), HET (n=12), and SC-HET (n=12) mice. (C) Grip strength was evaluated from P23 to P33 for SC-Sp-SMA mice (n=7) and SC-HET (n=12) mice. *P*=0.063, SC-Sp-SMA mice vs their heterozygous littermate mice (termed SC-HET) at P33. (D) The genotypes of SC-SMA mice. The deletions are indicated by a red dashed line. (E) NMJ innervation patterns of FDB muscles are indicated with NF-M^+^ motor nerves and BTX^+^ endplates. Scale bar, 50 μm. (F) SC-Sp-SMA mice could produce offsprings. (G) NHEJ and lifespan >400 days efficiency in offsprings (F1) of SC-Sp-SMA mice. (H) Quantification of gems per 100 motor neurons in SMA (n=3), F1-SC-Sp-SMA (n=3) and SC-HET (n=3) mice. (***) *P*<0.001, unedited SMA mice vs F1-SC-Sp-SMA mice at P9. (I) α-MNs in the spinal cord L1~L2 ventral horn were counted in SMA (n=6), F1-SC-Sp-SMA (n=7) and SC-HET(n=6) mice. (*) *P*<0.05, unedited SMA mice vs F1-SC-Sp-SMA mice at P9. (J) Quantification of the innervated NMJs in SMA(n=3), F1-SC-Sp-SMA (n=3) and SC-HET(n=4) mice. Data are presented as mean ± SD. One-way ANOVA.

**Table S1. Off-target analysis in SMA iPSCs by Digenome-seq.**

| sgRNA | Predicted off-target sites by Cas-OFFinder | | Genome-wide captured sites from Digenome-seq | Validated sites of Deep sequencing |
| --- | --- | --- | --- | --- |
|  | Number of mismatches | Number of sites | Total number | Number of validated sites |
| sgRNA1 in SMA iPSCs | 0 | 1 | 1 | 1 |
|  | 1 | 0 | 0 | 0 |
|  | 2 | 1 | 1 | 0 |
|  | 3 | 28 | 0 | 0 |
|  | 4 | 281 | 0 | 0 |
|  | 5 | 1000 | 0 | 0 |
|  | 6 | 1000 | 0 | 0 |
|  | 7 | 1000 | 0 | 0 |
|  | 8 | 1000 | 0 | 0 |
| sgRNA4 in SMA iPSCs | 0 | 1 | 1 | 1 |
|  | 1 | 1 | 0 | 0 |
|  | 2 | 2 | 1 | 0 |
|  | 3 | 23 | 10 | 0 |
|  | 4 | 265 | 13 | 0 |
|  | 5 | 1000 | 4 | 0 |
|  | 6 | 1000 | 0 | 0 |
|  | 7 | 1000 | 0 | 0 |
|  | 8 | 1000 | 0 | 0 |

**Table S2. Genotypes of SC-Sp-SMA.**

| **SC-Sp-SMA** | **Sequence(5'→3')** |  | **Frequence** | **Lifespan**  **(days)** |
| --- | --- | --- | --- | --- |
| #5 | TTCCTTAAATTAAGGAGTAAGTCTGCCAGCAT**CTTT**TATGAAAGTGAATCTTACTTTTGTAAAACTTTATGGTTTGT | +4bp | 4/10 | >500 |
|  | TTCCTTAAATTAAGGAGTAAGTCTGCCAGCA**CACTTAC**TTATGAAAGTGAATCTTACTTTTGTAAAACTTTATGGTTTGT | +7bp | 3/10 |  |
|  | TTCCTTAAATTAAGGAGTAAGTCTGCCAGCA**CACCCGGC-**TATGAAAGTGAATCTTACTTTTGTAAAACTTTATGGTTTGT | +8bp;-1bp | 1/10 |  |
|  | TTCCTTAAATTAAGGAGTAAGTCTGCCAGCA**--**ATGAAA**CATTTTTTTTGTAAACATTTTTG-----------**TTTTGTAAAACTTTATGGTTTGT | -2bp;+23bp;-11bp | 1/10 |  |
|  | TTCCTTAAATTAAGGAGTAAGTCT**----------------------------------------------------------------------------------------------------** | big deletion | 1/10 |  |
| #6 | TTCCTTAAATTAAGGAGTAAGTCTG**(503bp)------**TTATGAAAGTGAATCTTACTTTTGTAAAACTTTATGGTTTGT | +503bp;-8bp | 1/10 | >500 |
|  | TTCCTTAAATTAAGGAGTAAGTCTGCCAGCA**CACCCGGC-**TATGAAAGTGAATCTTACTTTTGTAAAACTTTATGGTTTGT | +8bp;-1bp | 1/10 |  |
| #7 | TTCCTTAAATTAAGGAGTAAGTCTGCCAGCA**C**TTATGAAAGTGAATCTTACTTTTGTAAAACTTTATGGTTTGT | +1bp | 4/10 | 241 |
| #8 | TTCCTTAAATTAAGGAGTAAGTCTGCCAGC**-**TTATGAAAGTGAATCTTACTTTTGTAAAACTTTATGGTTTGT | -1bp | 1/10 | >500 |
|  | TTCCTTAAATTAAGGAGTAAGT**---------------------------------**GAATCTTACTTTTGTAAAACTTTATGGTTTGT | -19bp | 3/10 |  |
| #9 | TTCCTTAAATTAAGGAGTAAGTCTGCCAGCA**GAATGAAAAATTTGAATGTGGA**TTATGAAAGTGAATCTTACTTTTGTAAAACTTTATGGTTTGT | +22bp | 1/10 | 224 |
|  | TTCCTTAAATTAAGGAGTAAGTCTGCCAGCA**GAATGAAAAATTTGAATGTGCATC**TTATGAAAGTGAATCTTACTTTTGTAAAACTTTATGGTTTGT | +24bp | 1/10 |  |
| #10 | TTCCTTAAATTAAGGAGTAAGTCTGC**(545bp)----**ATTATGAAAGTGAATCTTACTTTTGTAAAACTTTATGGTTTGT | +545bp;-4bp | 1/10 | 232 |
| #11 | TTCCTTAAATTAAGGAGTAAGTCTGCCAGCAT**---**GAAAGTGAATCTTACTTTTGTAAAACTTTATGGTTTGT | -3bp | 1/10 | >500 |
|  | TTCCTTAAATTAAGGAGTAAGTCTGCCAGCATT**TT**ATGAAAGTGAATCTTACTTTTGTAAAACTTTATGGTTTGT | +2bp | 3/10 |  |
| #12 | TTCCTTAAATTAAGGAGTAAGTCTGC**---------**TTATGAAAGTGAATCTTACTTTTGTAAAACTTTATGGTTTGT | -5bp | 8/10 | >500 |
| #13 | TTCCTTAAATTAAGGAGTAAGTCTGCC**----**TTATGAAAGTGAATCTTACTTTTGTAAAACTTTATGGTTTGT | -2bp | 1/10 | 98 |
|  | TTCCTTAAATTAAGGAGTAAGTCT**--------**TATGAAAGTGAATCTTACTTTTGTAAAACTTTATGGTTTGT | -8bp | 1/10 |  |
|  | TTCCTTAAATTAAGGAGTAAGTCTG**-----------**AAAGTGAATCTTACTTTTGTAAAACTTTATGGTTTGT | -11bp | 1/10 |  |
|  | TTCCTTAAATTAAGGAGTAAGTCTGC**(545bp)----**ATTATGAAAGTGAATCTTACTTTTGTAAAACTTTATGGTTTGT | +545bp;-4bp | 1/10 |  |
| #14 | TTCCTTAAATTAAGGAGTAAGTCT**--------**TATGAAAGTGAATCTTACTTTTGTAAAACTTTATGGTTTGT | -8bp | 1/10 | 253 |
|  | TTCCTTAAATTAAGGAGTAAGTCTGCCAGCA**GAATGAAAAATTTGAATGTGGA**TTATGAAAGTGAATCTTACTTTTGTAAAACTTTATGGTTTGT | +22bp | 3/10 |  |

**Table S3. Genotypes of SC-Sa-SMA.**

| **SC-Sa-SMA** | **Sequence(5'→3')** |  | **Frequence** | **Lifespan (days)** |
| --- | --- | --- | --- | --- |
| #3 | TTCCTTAAATTAAGGAGTAAGTCTGCCAGCATTAT**-**AAAGTGAATCTTACTTTTGTAAAACTTTATGGTTTGT | -1bp | 10/10 | 201 |
| #4 | TTCCTTAAATTAAGGAGTAAGTCTGCCAGCATT**T**ATGAAAGTGAATCTTACTTTTGTAAAACTTTATGGTTTGT | +1bp | 10/10 | >500 |
| #5 | TTCCTTAAATTAAGGAGTAAGTCTGCCAGCATTATG**---------**TGAATCTTACTTTTGTAAAACTTTATGGTTTGT | -4bp | 10/10 | >500 |
| #6 | TTCCTTAAATTAAGGAGTAAGTCTGCCAGCATTATG**---------**TGAATCTTACTTTTGTAAAACTTTATGGTTTGT | -4bp | 10/10 | >500 |
| #7 | TTCCTTAAATTAAGGAGTAAGTCTGCCAGCATTAT**AT-**AAAGTGAATCTTACTTTTGTAAAACTTTATGGTTTGT | +2bp;-1bp | 10/10 | 190 |
| #8 | TTCCTTAAATTAAGGAGTAAGTCTGCCAGCATTATGAA**------------**TCTTACTTTTGTAAAACTTTATGGTTTGT | -6bp | 10/10 | >500 |
| #9 | TTCCTTAAATTAAGGAGTAAGTCT**------------------------------------------**TACTTTTGTAAAACTTTATGGTTTGT | -23bp | 10/10 | >500 |

**Table S4. Off-target analysis in SMA mice by Digenome-seq.**

| sgRNA | Predicted off-target sites by Cas-OFFinder | | Genome-wide captured sites from Digenome-seq | Validated sites of Deep sequencing |
| --- | --- | --- | --- | --- |
|  | Number of mismatches | Number of sites | Total number | Number of validated sites |
| sgRNA1 in SMA mice | 0 | 1 | 1 | 1 |
|  | 1 | 0 | 0 | 0 |
|  | 2 | 1 | 0 | 0 |
|  | 3 | 17 | 0 | 0 |
|  | 4 | 187 | 0 | 0 |
|  | 5 | 1632 | 0 | 0 |
|  | 6 | 11605 | 0 | 0 |
|  | 7 | 64769 | 0 | 0 |
|  | 8 | 300029 | 0 | 0 |
| sgRNA5 in SMA mice | 0 | 1 | 1 | 1 |
|  | 1 | 0 | 0 | 0 |
|  | 2 | 1 | 0 | 0 |
|  | 3 | 17 | 0 | 0 |
|  | 4 | 187 | 0 | 0 |
|  | 5 | 1632 | 1 | 0 |
|  | 6 | 11605 | 0 | 0 |
|  | 7 | 64769 | 0 | 0 |
|  | 8 | 300029 | 0 | 0 |
