# Supplementary material for: Disruption of splicing-regulatory elements using CRISPR/Cas9 to rescue spinal muscular atrophy in human iPSCs and mice
Source: Natl Sci Rev. 2019 Sep 3;7(1):92–101. doi: 10.1093/nsr/nwz131 (PMC8446915; doi:10.1093/nsr/nwz131)
Supplement: nwz131_Supplemental_Files [file nwz131_supplemental_files.zip › nwz131_Supplementary_Methods.docx]

**METHODS**

**Plasmid construction**

The pU6-sgRNA-pEF1α-spCas9-2A-EGFP-expressing plasmid was subcloned from plasmids px458 (Addgene, #48138) and pAAV-EF1a-DIO-mCherry-WPRE-pA (Addgene, #37083). Four guide RNAs were designed to specifically target ISS-N1 and ISS+100, respectively, and were cloned into the aforementioned expression plasmid using T4 ligation.

| **Name** | **Primer Sequences (5’-3’)** | **Target** |
| --- | --- | --- |
| sgRNA1 | Forward-caccGAAGATTCACTTTCATAATGC | ISS-N1 |
|  | Reverse-aaacGCATTATGAAAGTGAATCTTC |  |
| sgRNA2 | Forward-caccGCTTACTTTTGTAAAACTTTA | ISS-N1 |
|  | Reverse-aaacTAAAGTTTTACAAAAGTAAGC |  |
| sgRNA3 | Forward-caccGTTGTAAAACTTTATGGTTTG | ISS-N1 |
|  | Reverse-aaacCAAACCATAAAGTTTTACAAC |  |
| sgRNA4 | Forward-caccGTCAGATGTTAGAAAGTTGAA | ISS+100 |
|  | Reverse-aaacTTCAACTTTCTAACATCTGAC |  |

**T7EI assay**

For assessing the activity of different sgRNAs, genomic DNA was extracted on day 2 or 3 after transient transfection using Quick DNA Extraction Solution (0.1% Triton X-100, 0.1% Tween 20 and 4 μg/ml proteinase K) at 56℃ for 20min and 95℃ for 5min. The targeted regions were amplified with the primers below. The T7EI assay was performed according to the manufacturer’s instructions. Briefly, 500 ng PCR products purified by a Gel Extraction Kit (OMEGA) were denatured and reannealed in Buffer 2 (NEB) by heating at 95°C for 5min and slow step-wise cooling to 25°C. Then 0.5 μl T7 Endonuclease I (T7EI) (NEB) was added and incubated at 37°C for 1h. The products were separated on a 2.5% agarose gel. The fraction of cleavage intensity was measured using ImageJ (NIH, MD, USA). Indel occurrence can be estimated according to previously described protocol [18].

| **Name** | **Primer Sequences (5’-3’)** | **Target** |
| --- | --- | --- |
| Surveyor-1 | Forward-AGACTATCAACTTAATTTCTGATC | ISS-N1 and ISS+100 |
| Surveyor-2 | Reverse-TGCCTAGGTTATCCCATATCACA |  |

**Human iPS cell reprogramming and spinal motor neuron differentiation**

Skin fibroblasts from an SMA patient (GM00232, Coriell Institute) and healthy control fibroblasts were reprogrammed to human induced pluripotent stem (iPS) cells using Sendai virus (Thermo Scientific) according to the manufacturer’s instructions. SMA-2 iPS cells, established with episomal vectors, were obtained from Coriell Cell Repositories (GM24468).

All iPS cell lines were maintained on hESC qualified Matrigel (Corning) in Nova medium (Nuwacell Biotechnology CO., Ltd). iPS cells were differentiated into spinal motor neurons with our modified protocol [26]. In brief, confluent iPS cells were detached and differentiated to embryoid bodies (EBs) in a neural medium consisting of DMEM/F12, Neurobasal medium at 1:1, 0.5$\times$N2, 0.5$\times$B27, 0.1mM ascorbic acid, 1$\times$nonessential amino acids,1$\times$glutamax and 1$\times$penicillin/streptomycin (all from Thermo Scientific) in the presence of CHIR99021 (3 μM, Stemgent), SB431542 (2 μM, Torcris), and LDN 193189 (0.3 μM, Stemgent). On day 2, the EBs were treated with RA (0.1 μM, Sigma) and SAG (0.5 μM, Millipore) for neuroepithelia (NE) induction. On day 4, the neuralized EBs were suspended in the same medium, but without SB431542, LDN193189, or CHIR99021. To generate synchronized postmitotic neurons, the dissociated neurospheres were plated on laminin substrate and treated from day 10 to 12 with DAPT (10 μM).

**Generation of clonal iPS cell lines**

For transfection, iPSCs were dissociated using Accutase (Thermo Scientific) and transfected in suspension with the sgRNA1 and sgRNA4 plasmids using nucleofection (Bio-Rad), then replated in a Matrigel-coated 10-cm dish (Corning) with Essential 8 (E8) medium in the presence of Y27632 (2 μM, Tocris) and 1$\times$penicillin/streptomycin. After 2 days, EGFP^+^ cells were sorted using a fluorescence activated cell sorting sorter (Beckman) into a Matrigel-coated 10-cm dish at a low density of 5$\times$10^4^ cells in the same medium. After one week, individual colonies were manually scraped and expanded for genotyping analysis.

**Genotyping analysis of cell lines**

The genomic DNA of cells was extracted using a TIANamp Genomic DNA Kit (TIANGEN). The targeted regions were amplified with the Surveyor assay primers. To determine the specific genotypes, PCR products were cloned into pMD-19 T-vectors (Takara) and then screened using Sanger sequencing. 25 to 35 clones for per cell line were picked to calculate the editing ratio. Considering these cell lines were all single clones from edited SMA-2 iPSCs with 3 SMN2 copies, we defined the editing rate of ≥ 30% as one copy editing, ≥60% as two copies editing, and ≥90% as three copies editing.

**RNA extraction and RT-PCR**

Total RNA from cells or mice tissues was extracted with TRIzol reagent (Invitrogen). An aliquot containing 0.5-μg of total RNA was reverse-transcribed into cDNA using HiScript II Q RT SuperMix kit (Vazyme) according to the manufacturer’s instructions. Reverse transcription PCR (RT-PCR) was performed with the primers detailed below. The resulting products were visualized on 2.5% agarose gels using a UV transilluminator (Tanon).

| **Name** | **Primer Sequences (5’-3’)** | **Target** |
| --- | --- | --- |
| RT-PCR-1 | Forward-TCTCTTGATGATGCTGATGC | ISS-N1 and ISS+100 |
| RT-PCR-2 | Reverse-TCTGATCGTTTCTTTAGTGGTGTC |  |

**Western blot**

The cells or mice tissues were lysed in radioimmunoprecipitation assay (RIPA) buffer (Beyotime) supplemented with 1× protease inhibitor cocktail (Biotool). Protein extracts were separated on 10% SDS-PAGE gels and immunoblotted with primary antibodies, as follows: rabbit anti-SMN antibody (1:500, Santa Cruz) and mouse anti-β-tubulin antibody (1:5000, Beyotime). HRP-conjugated secondary antibodies (1:1,000) were used to detect primary antibodies, and proteins were visualized with a BeyoECLmoon kit (all from Beyotime).

**Karyotype analysis and teratoma assays**

Standard Giemsa-banding chromosomeanalysis was carried out for each iPSC clone. To examine the developmental potential of iPSC clones *in vivo*, cells grown on Matrigel in E8 medium were collected by EDTA treatment, and injected into hind limb muscles of 6-week-old immunocompromised severe combined immune deficient (SCID)-beige mice (approximately 3 x 10^6^ cells for each site). After nine weeks, teratomas were dissected and fixed in 4% paraformaldehyde. Samples were embedded in paraffin and processed with hematoxylin and eosin (HE) staining.

**Alkaline phosphatase (AP) staining and immunocytochemistry**

Cells were fixed in 4% paraformaldehyde (PFA) for 15 min. AP staining (Roche) was carried out according to the manufacturer's recommendations. For immunocytochemistry, cells were permeabilized with 0.2% Triton-X-100 and blocked with 10% donkey serum, then incubated with the primary antibodies detailed below. Cellular nuclei were counterstained with DAPI (1:1,000, Sigma). Images were captured by a Leica TCS SP8 confocal system. Image-J software was used for further quantification of the cell population. Cell counting was performed by a person blind to the experiment and replicated in five random visual fields from three independent experiments.

| **Antibody** | **Isotype** | **Source** | **Catalogue Number** | **Dilution** |
| --- | --- | --- | --- | --- |
| Nanog | Goat IgG | R&D | AF1997 | 1:1,000 |
| OCT4 | Mouse IgG | Millipore | MAB4401 | 1:1,000 |
| SOX2 | Goat IgG | R&D | AF2018 | 1:1,000 |
| Tra-1-60 | Mouse IgG | Millipore | MAB4360 | 1:1,000 |
| SSSE4 | Mouse IgG | Millipore | MAB4304 | 1:400 |
| SOX1 | Goat IgG | R&D | AF3369 | 1:1,000 |
| Nestin | Rabbit IgG | Millipore | ABD69 | 1:1,000 |
| Olig2 | Rabbit IgG | Abcam | AB109186 | 1:300 |
| SMN | Rabbit IgG | Santa Cruz | SC15320 | 1:75 |
| TUJ1 | Mouse IgG | COVANCE | MMS-489P | 1:10,000 |
| TUNEL | NA | Beyotime | C1090 | 1:10 |

**Mice**

All of the mouse work was approved by the Institutional Animal Care and Use Committee of the Institute of Neuroscience, Chinese Academy of Sciences. The original breeding pair of heterozygous BH mice (*Smn*^+/-^) and HF-III mice (*Smn*^-/-^; *SMN2*^tg/tg^, stock number: 005058) in the FVB background were obtained from the Jackson Laboratory. HF-III mice carry four *SMN2* copies and do not develop a severe SMA phenotype, but gradually present necrotic ears and a short tail. The BH mice were crossbred to the HF-III mice to generate severe SMA mice (*Smn*^-/-^; *SMN2*^tg/-^). In each litter, half of the offspring were severe SMA mice and the other half were heterozygous controls (*Smn*^+/-^; *SMN2*^tg/-^). Primers of tri-primer-PCR for SMA mice genotyping are listed below.

| **Name** | **Primer Sequences (5’-3’)** |
| --- | --- |
| TP-PCR-1 | Forward-ATAACACCACCACTCTTACTC |
| TP-PCR-2 | Reverse- AGCCTGAAGAACGAGATCAGC |
| TP-PCR-3 | Reverse-GTAGCCGTGATGCCATTGTCA |

**Production of Cas9 mRNA and sgRNA**

The T7 promoter was added to the Cas9 coding region. sgRNA, obtained via PCR amplification from the px260 and px601 plasmids (Addgene; primers are listed below) and subsequent purification, were used as the template for *in vitro* transcription using a MEGAshortscript T7 Transcription Kit (Thermo scientific). T7-Cas9 PCR products were purified and used as the template for *in vitro* transcription using a mMESSAGE mMACHINE T7 ULTRA kit (Thermo scientific). T7-sgRNA PCR products were purified and used as the template for *in vitro* transcription using a MEGA clear kit (Thermo scientific).

| **Name** | **Primer Sequences (5’-3’)** |
| --- | --- |
| SpCas9 | Forward -TAATACGACTCACTATAGGGAGATTTCAGGTTGGACCGGTG |
|  | Reverse-GACGTCAGCGTTCGAATTGC |
| SaCas9 | Forward – TAATACGACTCACTATAGGGCTAACTACCGGTGCCACCATG |
|  | Reverse- GGTACCTCCCCAGCATGCCT |
| sgRNA1 | Forward –TAATACGACTCACTATAGGGAAGATTCACTTTCATAATGC |
|  | Reverse-AAAAGCACCGACTCGGTGCC |
| sgRNA4 | Forward-TAATACGACTCACTATAGGGTCAGATGTTAGAAAGTTGAA GTTTTAGAGCTAGAAATAG |
|  | Reverse-AAAAGCACCGACTCGGTGCC |
| sgRNA5 | Forward-TAATACGACTCACTATAGGGAAGTCTGCCAGCATTATGAAA |
|  | Reverse- AAAAATCTCGCCAACAAGTTG |

**Zygote injection and embryo transplantation**

Heterozygous female BH mice (*Smn*^+/-^) or HF-III mice (*Smn*^-/-^; *SMN2*^tg/tg^) were super-ovulated and mated to HF-III males or heterozygous BH males. The fertilized embryos were collected for injection and transplantation. Briefly, Cas9 mRNA (50 ng/μl) and sgRNA (100 ng/μl) were injected into the cytoplasm of fertilized embryos with well-recognized pronuclei. The injected zygotes were incubated in KSOM overnight until the two-cell stage, and were then transferred into the oviducts of pseudo-pregnant ICR female mice at 0.5 dpc.

**Genotyping analysis of germline-corrected mice**

The genomic DNA of mice tails was extracted using TIANamp Genomic DNA Kits (TIANGEN). The targeted regions were amplified with Surveyor assay primers. To determine the specific genotypes, PCR products were cloned into the pMD-19 T-vector (Takara), and then screened using Sanger sequencing. 10 to 15 clones for per cell line were picked, assessed, and used to calculate the editing ratio.

**Behavioral analysis**

The righting reflex and gripping tests were performed as previously described [31]. Briefly, for the righting-reflex analysis, mice were placed on their back and the time to upright themselves were calculated. Each mouse was tested 3 times with 1-min intervals. The average time taken was recorded. For the gripping test, mice were allowed to grasp a triangular bar (grip-strength meter, Columbus Instrument) with their forelimbs and hindlimbs when dragged horizontally. The test was repeated 3 times for each mouse, and the highest value was recorded.

**Assessment of spinal motor neuron pathology in mice**

Mice were euthanized at P9, perfused with 1$\times$phosphate buffer solution (PBS) followed by 4% PFA. Spinal cords were removed and fixed in 4% PFA for 2-4 hours, then allowed todehydrate in 30% sucrose overnight. The lumbar (L1-L2) spinal cords were sectioned at 15μm and standard immunofluorescence staining was carried out. Motor neurons and Gemini bodies were stained with goat anti-choline acetyltransferase (ChAT) (1:300, Millipore, AB144P) and mouse anti-SMN (1:300, Millipore, 05-1532), respectively. Motor neurons were counted in a total of 3-5 mice from each group, assessing 10 serial sections from each mouse; each section was at least 100 microns apart.

**Assessment of neuromuscular junction (NMJ) morphology in mice**

Mice were sacrificed at P9, perfused with 1$\times$phosphate buffer solution (PBS) followed by 4% PFA. Flexor digitorumbrevis (FDB) muscles were used to examine NMJ innervation patterns. Whole FDB muscles were teased gently into layers with 5-10 fibers, and motor nerves were labeled with a rabbit polyclonal antibody against neurofilamentmedium (NF-M) (1:200, Millipore, AB1987) and acetylcholine receptors (AChR) with α-bungarotoxin (BTX) (Molecular Probes, 1:500, B13423). The proportions of innervated or denervated endplates identified by both AChR clusters and nerve terminals were calculated.

**Digenome-seq off-target analysis**

Genomic DNA was extracted from iPSCs or mice tails with TIANamp Genomic DNA Kits (Tiangen) according to the manufacturer's instructions. Cas9 ribonucleoproteins (1 μg) and sgRNA (1 μg) were pre-incubated at room temperature for 10 min to form RNP complexes. Genomic DNA (4 μg) was incubated with RNP complexes in a 20-μl reaction buffer for 3 h at 37°C. Then the genomic DNA was purified with a DNA Purification Kit (Tiangen) after RNase A (100 μg/ml) was added to remove sgRNA at 37°C for 30 min.

Libraries were subjected to whole-genome sequencing (WGS) using an Illumina HiSeq X Ten sequencer, at a sequencing depth of 15$\times$. Qualified reads were aligned to the human reference genome (hg19) or mouse reference genome (mm10) by Isaac aligner with the following parameters: base quality cutoff, 15; keep duplicate reads, yes; variable read length support, yes; realign gaps, no. The mapped bam files were sorted and indexed with Picard tools (v2.3.0). DNA cleavage sites were identified computationally using Digenome-seq2 (<https://github.com/chizksh/digenome-toolkit2>) with a depth cutoff of 0. The resulting multiplex Digenome-captured sites are listed in Supplementary Table 1 and 4. Then, we designed primers flanking the Digenome-captured off-target sites to perform genomic PCR for these three clones and then underwent deep sequencing.

**RNA-seq off-target analysis**

High-throughput mRNA sequencing (RNA-seq) was carried out using Illumina Hiseq with ~70 million reads for each sample. FastQC (v0.11.3) and Trimmomatic (v0.36) were used for quality control. Qualified reads were mapped to the reference genome (Ensemble GRCh38) using STAR (v2.5.2b) in 2-pass mode with the parameters implemented by the ENCODE project. Picard tools (v2.3.0) was then applied to sort and mark duplicates of the mapped BAM files. The refined BAM files were subject to split reads that spanned splice junctions and local realignment, base recalibration, and variant calling were performed with GATK (v3.5). To identify variants with high confidence, we filtered clusters of at least 5 SNVs that were within a window of 35 bases and retained variants with base quality scores >25, mapping quality scores >20, Fisher Strand values (FS > 30.0), Qual By Depth values (QD < 2.0), and sequencing depth > 20.

***Deep sequencing analysis***

A previously described pipeline, Hi-TOM, was used for the deep sequencing data analysis [50]. Briefly, two rounds of PCR amplification for ISS-N1 or ISS+100 were performed using the primers detailed below. Equal amounts of the PCR products were mixed and then purified. Sequencing was next performed with the Illumina HiSeq 3000 platform. The resulting NGS data was uploaded to the Hi-TOM platform for high-throughput mutation sequence decoding (http://www.hi-tom.net/hi-tom/).

| **Name** | **Primer Sequences (5’-3’)** | **Target** |
| --- | --- | --- |
| Hi-TOM-1F | ggagtgagtacggtgtgcATCAAAAAGAAGGAAGGTGC | ISS-N1 |
| Hi-TOM-1R | gagttggatgctggatggCATTTGTTTTCCACAAACCA |  |
| Hi-TOM-2F | ggagtgagtacggtgtgcTATGGTTTGTGGAAAACAAA | ISS+100 |
| Hi-TOM-2R | gagttggatgctggatggCCTTTTATCTAATAGTTTTGGC |  |

**Statistical analysis**

Data are shown as means ±SDs, if not indicated otherwise. Kaplan-Meier survival data were analyzed with the GraphPad Prism. One-way ANOVA were used to assess statistical significance. Differences were considered statistically significant when P values were less than 0.05 (*), 0.01 (**), 0.001 (***) or 0.0001 (****). n.s., not significant.
